# Socio-economic and age variations in response to supermarket-led checkout food policies: a repeated measures analysis

**DOI:** 10.1186/s12966-018-0755-4

**Published:** 2018-12-05

**Authors:** Katrine Ejlerskov, Stephen J. Sharp, Martine Stead, Ashley J. Adamson, Martin White, Jean Adams

**Affiliations:** 1 MRC Epidemiology Unit & Centre for Diet and Activity Research, University of Cambridge, Cambridge, UK; 2 Institute of Social Marketing, University of Stirling, Stirling, UK; 3 Institute of Health & Society and Human Nutrition Research Centre, Newcastle University, Newcastle upon Tyne, UK

**Keywords:** Inequalities, Public health, Diet, Policy, Supermarket

## Abstract

**Background:**

Dietary inequalities between population groups are common with older and more affluent individuals tending to have healthier diets. Differential responses to health interventions may exacerbate inequalities. Changing what foods are displayed at supermarket checkouts is one intervention that has the potential to change diets. The aim of this study was to assess whether differences in purchases of common checkout foods from supermarkets with different checkout food policies varied according to age group and social grade.

**Methods:**

We analysed annual household purchase data for 2013–17 from nine leading UK supermarkets, split according to age of the main household shopper and household social grade. Checkout food policies were categorised as clear and consistent, vague or inconsistent, and none. Policies were heterogeneous but all included removal of confectionery and/or chocolate from checkouts. Mixed effects linear regression models were used to assess differences in purchases of common checkout foods (sugary confectionery, chocolate and potato crisps) by checkout food policy and whether these varied by age group or occupational social grade.

**Results:**

Relative to supermarkets with no checkout food policy, 14% (95% CI: 4–22%) fewer purchases of common checkout foods per household per percentage market share were made in supermarkets with a clear and consistent policy. Adjusted mean numbers of purchases were higher in older age groups than the youngest, but there were no differences between the highest and other social grades. There were significant interactions between checkout food policy and both age group and social grade. In supermarkets with clear and consistent policies, 23% (6–36%), 20% (2–34%), and 23% (7–37%) fewer purchases were made in age groups 45–54, 55–64 and 65+ years respectively, compared to all groups combined. In supermarkets with clear and consistent policies, there were 21% (4–35%), 26% (9–39%) and 21% (3–35%) fewer purchases made by households in the highest two and lowest social grades respectively, compared to all groups combined.

**Conclusions:**

Households with older main shoppers and those in the most and least affluent social grades may be most responsive to supermarket checkout food policies. As older and more affluent groups tend to have healthier diets overall, it is unlikely that supermarket checkout food policies contribute to greater dietary equity.

## Background

Unhealthy dietary habits, obesity and diet-related chronic diseases are often found to exhibit a socioeconomic gradient, with higher prevalence among those of lower socio-economic position [1–3]. Similar inequalities have been seen by age, sex and ethnicity [2, 3].

Population interventions have been proposed as a stronger approach to prevention than interventions targeting those at high-risk [4]. However, some population interventions may have differential effects by socio-economic position, exacerbating existing inequalities in health. In particular, interventions characterised as high-agency, which rely on individuals’ using their personal resources to benefit (such as labelling or education), may be less effective in less affluent groups [5, 6]. In contrast, lower-agency population interventions that require recipients to use few of their personal resources to benefit (such as nutrient fortification of foods or marketing controls) are thought to be less likely to exacerbate existing inequalities [5–7].

Many UK supermarkets have introduced policies to improve the nutritional quality of the food displayed at their checkouts [8]. Checkout food policies can be characterised as low-agency population interventions as the majority of shoppers are exposed and they reduce the opportunity for impulse purchases without relying on shoppers to make any conscious changes to benefit. Current supermarket checkout food policies in the UK are voluntary commitments made by supermarkets without any particular government prompt or involvement. Although there is no systematic assessment of why supermarkets made these commitments, they may be a response to consumer pressure and advocacy group campaigns [9]. Whilst the nature of checkout food policies differs between UK supermarkets, all mention removal of confectionery and/or chocolate from checkouts with varying level of detail on other products to be removed, or introduced. Policies also vary in the consistency with which they apply to all checkouts within a supermarket group [8]. It is unknown what proportion of total food is purchased from the checkout.

The supermarket checkout area is recognised as an important point-of-purchase location with high levels of impulse buys [10] and high proportions of less healthy foods [11–13]. Recently, we found fewer less healthy foods at checkout in supermarkets with checkout food policies, particularly when policies were clear and consistent [8]. Further, we found that implementation of checkout food policies was associated with a decrease in purchases of less healthy common checkout foods compared to what would have been expected had policies not been implemented (Ejlerskov, 2018). However, it is not known if checkout food policies have differential impacts on purchasing by socio-economic position or other shopper characteristics. The aim of this study was to assess whether differences in purchases of common checkout foods from supermarkets with different checkout food policies vary according to age group or socio-economic position. More generally, this allowed us to test the hypothesis that low-agency population interventions, such as supermarket checkout food policies, are unlikely to exacerbate existing inequalities in diet.

## Methods

We conducted a repeat measures analysis of data from a commercial household consumer panel on purchases from nine leading UK supermarket groups (referred to throughout as ‘supermarkets’) from 2013 to 17. Annual purchase data was split according to age of the main household shopper and household social grade (a measure of socio-economic position) to assess whether associations between supermarket checkout food policies and purchases differed by age or social grade. We focused on age and social grade as this information was available, whilst data on, for example, ethnicity and gender of main household shopper was not.

### Included supermarkets

The nine included supermarkets were: Aldi, Asda, Coop, Lidl, M&S, Morrisons, Sainsbury’s, Tesco, and Waitrose, which together represent more than 90% of the total UK grocery market share [14]. Excluded supermarkets were either highly specialised (with a predominant focus on frozen foods), online only, or smaller franchise stores. As our intention was to explore the differential impacts of checkout food policies on purchases, rather than ‘name and shame’ particular supermarkets, supermarkets are anonymized throughout.

### Checkout food policies in included supermarkets

As previously described [8], we obtained information on supermarket checkout food policies from supermarket annual reports, webpages and press releases. If we could not find the information we needed via these sources, we contacted supermarkets’ customer services. As a last resort, we used information from secondary sources such as newspapers. Also, as previously described [8], supermarkets were categorised according to the content of their checkout food policies: clear and consistent policies (provide clear and detailed information on which specific products should be removed and introduced, and which apply consistently to all checkouts in a supermarket); vague or inconsistent policies (provide non-specific information on which products should be removed or introduced; or policies that do not apply consistently to all checkouts); and no policy. One supermarket was split according to format (convenience stores versus larger stores) as there were different policies in the two formats. Thus there was a total of ten supermarkets in the analyses. In several cases, supermarkets introduced a checkout food policy during the study period 2013–17, meaning policy status changed over time.

### Definition of common checkout foods

We studied purchases of the most common, less healthy food categories found in the checkout area in a large survey of checkout food in 69 branches of the included supermarkets [8]: sugary confectionery, chocolate and potato crisps. As data in the purchase panel are not specific to where in-store products were selected from, we focused on single serving and smaller package sizes which are more likely to be found at checkouts [8]. Thus, we included single-unit package purchases of sugary confectionery of ≤225 g, chocolate of ≤125 g, and potato crisps of ≤50 g. Purchases of all three groups were collapsed for analyses and are termed ‘common checkout foods’ throughout.

### Outcome variable: Purchases of common checkout foods

Supermarket-specific data on purchases of common checkout foods was extracted from Kantar Worldpanel’s “Take home” panel dataset [15]. The “Take home” panel is a large commercial household panel of approximately 30,000 UK households. New potential households are recruited via postal invitation and a follow up phone call. Participating households record all food and beverage purchases, from any store, brought into the home using electronic scanners; household data is collected at recruitment. Information captured includes purchase store name, product line and package size. This allowed us to isolate purchases of specific products from specific stores within the panel. The panel is broadly representative of the UK in terms of region, occupational social grade, age of main shopper and number of children in the household. Households receive monetary incentives for taking part and are subject to quality control procedures. Most households stay in the panel for 2–3 years. Information from this panel has been found to reflect that from the Living Costs and Foods Survey – a government funded cross-sectional household consumption survey [16].

We obtained data on the number of annual packages of common checkout foods purchased, from 2013 (when this data became available) to 2017 (when we extracted it), weighted and uplifted by Kantar Worldpanel to represent all UK households (*n* = 27,385,050 households).

### Explanatory variables: Age of main household shopper and household social grade

Age of the main household shopper was categorised as < 28 years, 28–34 years, 35–44 years, 45–54 years, 55–64 years and > 65 years. Household social grade was assigned based on the occupation of the highest household earner and categorised using the Market Research Society coding (AB: higher and intermediate managerial, administrative and professional occupations, C1: Supervisory, clerical and junior managerial, administrative and professional occupations; C2: Skilled manual workers: D: Semi-skilled and unskilled manual workers; and E: State pensioners, casual and lowest grade workers, unemployed with state benefits only) [17, 18].

### Covariates: Supermarket market share and customer base

Grocery market share differs between supermarkets and changes over time. To adjust for this in the analyses, we obtained data on monthly grocery market share for each supermarket from Kantar Worldpanel. This information was used to calculate mean annual market share. Where information on market share was missing (*n* = 2) we estimated this from supermarkets’ 2017 annual reports and used this 2017 data throughout.

Supermarkets also differ in terms of the characteristics of their customer base. Kantar provided us with information on the percentage of grocery spend made by shoppers in each social grade and age group for each supermarket in 2017 in order for us to adjust for the characteristics of supermarkets’ customer base in the analyses. This information was used to calculate ‘shopper mean social grade’ for each supermarket, which was a weighted mean grocery spend across social grades (social grade AB assigned a value of 5, C1 assigned a value of 4 and so on), using the proportion of grocery spend by each social grade in the particular supermarket as weights. Similarly, ‘shopper mean age’ for each supermarket was calculated, using the mid-range age within each age group, and the proportion of spend by each age group in the supermarket as weights.

### Data analyses

Annual purchases of common checkout foods per household from all supermarkets combined were calculated as the sum of annual purchases of common checkout foods across supermarkets divided by the number of households in each year (2015–17 data provided from Kantar; 2013–14 data from the Office for National Statistics [19]). The number of UK households in each age group and social grade for the years 2015–17 were provided by Kantar and 2015 distributions were used to calculate 2013 and 2014 values. This enabled annual purchases of common checkout foods per household according to age group and social grade to be calculated.

We estimated associations and interactions using mixed effects linear regression models. The outcome in these analyses was annual units of common checkout foods purchased per percentage market share. This allowed any change in custom associated with interventions to be taken into account and changes associated with implementation of checkout food policies in supermarkets with different markets shares to be more comparable. The outcome was log-transformed to improve model fit. The exposure was checkout food policy status, coded 0 in years where no policy was present, 1 in years where a vague or inconsistent policy was present for at least six months (that is implementation was in January–June or the policy was pre-existing), and 2 in years where a clear and consistent was present for at least six months. Observations of annual purchases were clustered within supermarkets. All models included year, shopper mean age and shopper mean social grade as fixed effects, and a random intercept for supermarket.

In model 1, the overall difference in purchases by checkout food policy status, and comparisons between years were estimated, adjusted for mean shopper age and mean shopper social grade. The multiplicative interaction between checkout food policy status and age group was tested in model 2, and between checkout food policy status and social grade in model 3 using the ‘lincom’ (linear combinations of estimators) command in Stata. These models were then used to calculate differences in purchases by checkout food policy status across age group and social grade, as well as adjusted mean numbers of purchases within each combination of policy and age group, or policy and social grade.

All analyses were performed using Stata/SE v14.2 [20].

Ethical approval was not required for these analyses of aggregate anonymised purchase data.

## Results

Of the ten supermarkets, six implemented a checkout food policy during the study period of 2013–2017 (Table 1), three of which were clear and consistent and three vague or inconsistent. Two supermarkets implemented a vague or inconsistent policy before the study period and two had no policy throughout the study period. Annual market share of supermarkets was relatively stable, while annual purchases of common checkout foods showed greater variation. Households shopping at supermarket 5 tended to be oldest and those at supermarket 3 youngest (Table 1). Supermarket 4 had the most, and supermarket 7 the least, affluent shoppers.

**Table 1:** Characteristics of included supermarkets

Supermarket	Checkout food policy		Annual market share 2013–17, median [bottom quartile; top quartil]	Annual purchases of common checkout foods (1000s) 2013–17, median [bottom quartile; top quartile]	Shopper mean social grade (5 = most affluent)	Shopper mean age (years)
	Category	Implementation, month year				
1	Clear and consistent	Jan 2014	3.9 [3.5;4.3]	60,179 [57,076;69,738]	3.43	52.6
2	Clear and consistent	Jan 2015	28.4 [28.0;28.9]	365,001 [357,128;384,010]	3.48	51.4
3	Clear and consistent	Jan 2015	5.4 [4.6;6.0]	65,961 [62,470;67,277]	3.45	49.7
4	Vague or inconsistent	Aug 2014	5.0 [4.8;5.0]	42,994 [39,928;43,912]	3.98	55.9
5	Vague or inconsistent	Sep 2015	3.1^a^	34,659 [33,192;37,160]	3.71	58.6
6	Vague or inconsistent	Feb 2016	10.9 [10.3;11.1]	140,404 [131,333;140,866]	3.34	53.8
7	Vague or inconsistent	Unknown 2011	16.7 [15.0;17.2]	210,675 [186,455;212,429]	3.25	50.5
8a	Vague or inconsistent	Unknown 2004	15.7 [15.5;15.8]	142,153 [134,036;145,138]	3.64	53.5
8b	Absent	NA	0.8^a^	25,459 [21,338;25,668]	3.64	53.5
9	Absent	NA	6.2 [6.1;6.2]	134,700 [122,584;142,182]	3.32	55.4

^a^Market share estimated from 2017 annual reports throughout, so no variation present

### Differences in purchases of common checkout foods by checkout food policy status

Table 2 summarises the results of the mixed effects liner regression models.

**Table 2:** Differences in purchases of common checkout foods per household per percentage market share by checkout food policy status

		Ratio of geometric means (95% confidence intervals)		
Variable	Level	Model 1^a^	Model 2^b^	Model 3^c^
Checkout food policy (ref = none)	Vague or inconsistent	0.96 (0.87;1.06)	1.07 (0.90;1.27)	1.06 (0.89;1.27)
	Clear & consistent	0.86 (0.78;0.96)	1.16 (0.96;1.41)	0.79 (0.65;0.96)
Year (ref = 2013)	2014	0.97 (0.90;1;04)	1.01 (0.93;1.09)	0.97 (0.89;1.06)
	2015	1.06 (0.98;1.14)	1.13 (1.03;1.24)	1.07 (0.97;1.18)
	2016	1.10 (1.01;1.20)	1.20 (1.09;1.32)	1.11 (1.00;1.24)
	2017	1.12 (1.03;1.22)	1.19 (1.08;1.32)	1.13 (1.01;1.26)
Supermarket shopper mean age (years)		1.02 (0.92;1.12)	1.00 (0.90;1.11)	1.01 (0.91;1.11)
Supermarket shopper mean social grade (5 = most affluent)		0.48 (0.15;1.50)	0.39 (0.11;1.34)	0.36 (0.11;1.21)
Age (ref = <27y)	28-34y		1.26 (1.11;1.43)	
	35-44y		1.90 (1.67;2.17)	
	45-54y		2.22 (1.95;2.52)	
	55-64y		2.08 (1.82;2.37)	
	>64y		1.83 (1.60;2.08)	
Checkout food policy * age (ref = Vague or inconsistent, all age groups)	Vague or inconsistent, <28y		1.07 (0.90;1.27)	
	Vague or inconsistent, 28-34y		1.08 (0.91;1.28)	
	Vague or inconsistent, 35-44y		1.01 (0.85;1.20)	
	Vague or inconsistent, 45-54y		0.99 (0.83;1.17)	
	Vague or inconsistent, 55-64y		0.97 (0.82;1.15)	
	Vague or inconsistent, >64y		0.86 (0.72;1.01)	
Checkout food policy * age (ref = Clear & consistent, all age groups)	Clear & consistent, <28y		1.16 (0.96;1.41)	
	Clear & consistent, 28-34y		1.06 (0.88;1.29)	
	Clear & consistent, 35-44y		0.86 (0.71;1.05)	
	Clear & consistent, 45-54y		0.77 (0.64;0.94)	
	Clear & consistent, 55-64y		0.80 (0.66;0.98)	
	Clear & consistent, >64y		0.77 (0.63;0.93)	
Social grade (ref = AB)	C1			1.12 (0.98;1.28)
	C2			0.91 (0.80;1.04)
	D			0.99 (0.87;1.13)
	E			1.00 (0.87;1.13)
Checkout food policy * social grade (ref = Vague or inconsistent, all social grade groups)	Vague or inconsistent, AB			1.06 (0.89;1.27)
	Vague or inconsistent, C1			0.94 (0.79;1.12)
	Vague or inconsistent, C2			1.03 (0.87;1.23)
	Vague or inconsistent, D			0.96 (0.81;1.15)
	Vague or inconsistent, E			0.84 (0.71;1.01)
Checkout food policy * social grade (ref = Clear & consistent, all social grade groups)	Clear & consistent, AB			0.79 (0.65;0.96)
	Clear & consistent, C1			0.74 (0.61;0.91)
	Clear & consistent, C2			1.05 (0.86;1.29)
	Clear & consistent, D			0.96 (0.79;1.18)
	Clear & consistent, E			0.79 (0.65;0.97)

^a^Model 1 adjusted for year, supermarket shopper mean age, and supermarket shopper mean social grade. ^b^Model 2 adjusted for year, supermarket shopper mean age, supermarket shopper mean social grade, age group and multiplicative interaction between age group and checkout food policy status. ^c^Model 3 adjusted for year, supermarket shopper mean age, supermarket shopper mean social grade, social grade and multiplicative interaction between social grade and checkout food policy status

Model 1 in Table 2 shows differences in purchases of common checkout foods by checkout food policy status in all households, adjusted for year, supermarket shopper mean age and supermarket shopper mean social grade. Coefficients presented in Table 2 are ratios of geometric means of purchases, hence a value of 1.00 represents no difference in purchases.

Relative to supermarkets with no checkout food policy, 14% (95% confidence interval (CI): 4–22%) fewer purchases of common checkout foods per household per percentage market share were made from supermarkets with a clear and consistent checkout food policy (Table 2, model 1; ratio 0.86 (95%CI 0.78 to 0.96)). There was no evidence that purchases of common checkout foods from supermarkets with vague or inconsistent policies differed from those with no policy.

Model 1 also shows that annual purchases of common checkout foods increased by 10% (95% CI: 1–20%) in 2016 and 12% (3–22%) in 2017 compared to 2013. Supermarket mean shopper age and mean shopper social grade were not associated with annual purchases of common checkout foods.

### Age variations in purchases of common checkout foods by checkout food policy status

Model 2 in Table 2 shows that adjusted mean numbers of purchases of common checkout foods per household per percentage market share was higher in households with main shoppers in all age groups older than 28 years compared to those where the main shopper was < 28 years.

There was a significant interaction between age group and checkout food policy (*p* = 0.006). Compared to all households combined, in households with older main shoppers, fewer purchases of common checkout foods were made from supermarkets with a clear and consistent checkout food policy. Specifically, 23% (95% CI: 6–36%), 20% (2–34%), and 23% (7–37%) fewer purchases were made by households in age groups 45–54, 55–64 and 65+ years respectively. No differences in purchases of common checkout foods from supermarkets with vague or inconsistent policies were seen by age.

**Fig. 1:**
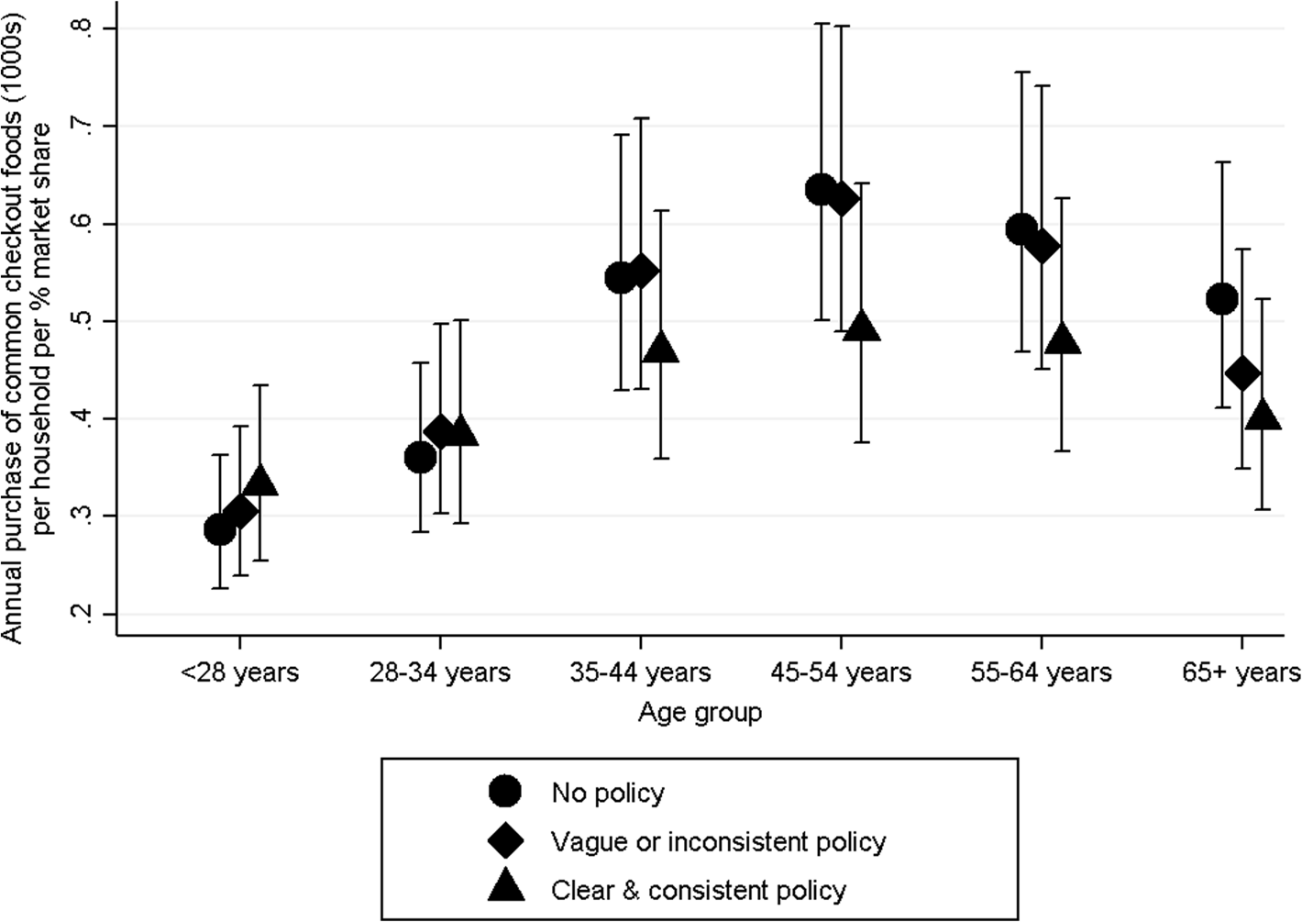
Adjusted mean (95%CI) annual purchases (1000s) of common checkout foods by household main shopper age

Thus, whilst purchases of common checkout foods were greater in older households, those in older groups purchased fewer common checkout foods from supermarkets with clear and consistent checkout food policies indicating that they may be more responsive to these policies (Fig. 1).

### Social grade variations in purchases of common checkout foods by checkout food policy status

Model 3 in Table 2 shows that adjusted mean numbers of purchases of common checkout foods per household per percentage market share did not vary in any social grade compared to grade AB.

There was a significant interaction between social grade and checkout food policy (*p* = 0.016). In households in social grades AB, C1 and E, fewer purchases of common checkout foods were made from supermarkets with a clear and consistent checkout food policy compared to all social grades combined. Specifically, 21% (95% CI: 4–35%), 26% (9–39%), and 21% (3–35%) fewer purchases were made in social grades AB, C1 and E respectively. No differences in purchases of common checkout foods from supermarkets with vague or inconsistent polices were seen by social grade.

**Fig. 2:**
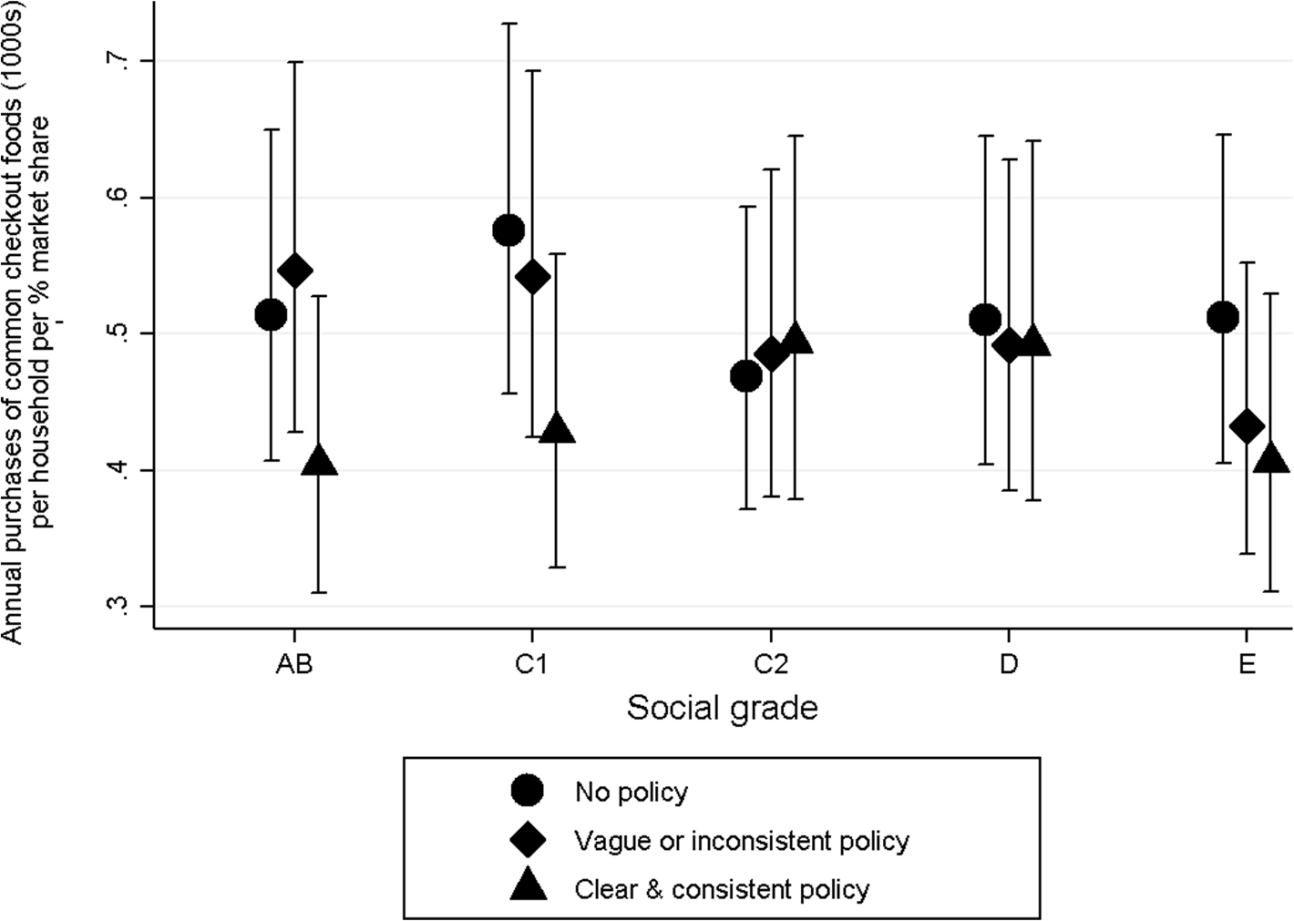
Adjusted mean (95%CI) annual purchases (1000s) of common checkout foods by household social grade

Thus, whilst there were no overall differences in purchases of common checkout foods by social grade, households in the top two and bottom social grade purchased fewer common checkouts foods from supermarkets with clear and consistent checkout food policies indicating they may be more responsive to these policies (Fig. 2).

## Discussion

To our knowledge, this is the first study to explore socio-demographic difference in purchases associated with a supermarket-led intervention in general, and supermarket checkout food policies in particular. Overall, 14% fewer purchases of common checkout foods per household per percentage market share were seen from supermarkets with clear and consistent checkout food policies compared to no policies, despite background increases in purchases of these foods over time. More purchases of common checkout foods were made by households with older main shoppers but there were no differences in purchases between the most affluent households and others. Households with older main shoppers and those in the most and least affluent social grades purchased significantly fewer common checkout foods from supermarkets with clear and consistent checkout food policies compared to all households combined, suggesting they may be more responsive to these policies. As older and more affluent groups tend to have healthier diets overall, it is unlikely that supermarket checkout food policies contribute to greater dietary equity overall.

### Strengths and limitations

The data used in this study are derived from a large, broadly representative panel. This is the most comprehensive data on UK food purchases brought into the home available at the product level, across the full year, and over time. As the panel data are multiplied up and weighted by Kantar to represent all UK households, our results are likely to be generalisable across the UK, but they may not be more widely generalisable. Analysing at the annual level provided the robustness required to split supermarket-specific data by household social grade and age group. However, it also made coding for when the checkout food policy status changed less accurate. Two supermarkets implemented vague or inconsistent checkout food policies before the start of the study period (when data became available). This meant we had no ‘baseline’ situation when all supermarkets had no policy. However, we could compare supermarkets with and without different policies at different time points.

Purchases that are eaten ‘on the go’ without entering the home are not recorded by the take-home panel used. This is likely to apply to some common checkout food purchases, although we do not know what proportion. However, unless the balance of take home to on the go purchases varies by checkout food policy status, this should not affect the differences in purchases by policy status we report. If there are age or social group variations in how much food is eaten ‘on the go’ versus brought home, this could be a source of bias.

We do not know where in store purchases were selected from. Restricting the analyses to smaller, single-serve package sizes increased the likelihood that purchases were from checkouts, albeit this may be neither sensitive nor specific. The impact of checkout food policies on overall diet is unknown and we do not know if those who purchased fewer common checkout foods compensated by purchasing other unhealthy items, other healthier items, other non-food items; or if they did not compensate. Although we adjusted for market share and characteristics of shoppers in different supermarkets, we cannot exclude selection bias in terms of which supermarkets chose to introduce checkout food policies.

Age group was determined as the age of the main household shopper. However, food brought into the house is not necessarily for the shopper. Classification of social grade was based on the occupation of the highest earner in the household [18]. Occupation is strongly associated with income, another factor often used to quantify socio-economic position [21]. Whilst resources are often shared within households, this is not always the case. Household social grade may not capture all socio-economic, and associated cultural, differences between households.

### Interpretation of results and comparison with other research

Overall we found greater purchases of common checkout foods in households with older compared to the youngest main food shoppers, but no differences in purchases from households in other social groups compared to the most affluent. This contrasts with previous research which demonstrated higher levels of self-reported non-grocery impulse shopping in shoppers with “some” college education, and those 18–39 years of age [22]. Different behavioural mechanisms may influence non-grocery versus grocery impulse purchasing and not all checkout food purchases may be impulse.

We found greater purchases of common less healthy checkout foods in households with older main shoppers, but no difference in purchases between the highest and other social grades. This contrasts to substantial population research showing that older [2] and more affluent [1, 2] people tend to have the healthiest diets. However, checkout food is likely to make up a small proportion of total diet. Inverse age and socioeconomic related differences in snacking behaviours have also been previously reported [23]. There is some evidence that older adults rely more on cognitive heuristics (i.e. simple thinking rules) than younger people, allowing them to make quicker decisions, but also making them more susceptible to distraction and irrelevant information and hence, perhaps, impulse shopping [24].

Clear and consistent checkout food policies were associated with greater decreases in purchases in households with older main shoppers. As older groups already have the healthiest diets, it is unlikely that clear and consistent checkout food policies ameliorate existing age-related inequalities in diet. Clear and consistent checkout food policies were also associated with greater decreases in purchases in households from the most and least affluent social grades. As the least affluent social grade includes retirees, this finding may be confounded by age. Given the most affluent groups have the healthiest diets, it is again unlikely that clear and consistent checkout food policies ameliorate existing socio-economic inequalities in diet.

Low-agency population interventions are typically believed to have equitable effects because they reach all those who are exposed to the intervention and require less agency from recipients [5–7]. An essential, but rarely stated, condition to achieve this equity is that the least healthy groups must be most likely to perform the behaviours targeted by interventions. Our findings suggest this is not the case in the context of purchases of common checkout food.

We found a stronger effect on purchasing of clear and consistent compared to vague or inconsistent checkout food policies. This may be explained by our previous finding that supermarkets with clear and consistent policies adhere to these better than those with vague or inconsistent policies [8].

The avoidance of pester power was the driving force behind two UK campaigns against ‘junk food’ at supermarket checkouts [9, 25, 26]. As we had no information on whether children were present when purchases were made, or whether households in the dataset included children, we were unable to explore whether checkout food policies were particularly effective in those shopping with, or for, children. This should be investigated in future research.

The effect size seen should be interpreted in the light of the overall frequency of purchasing of common less healthy checkout foods. Crude estimates from our data indicate that a total of 1381 million units of common less healthy checkouts foods were purchased from included supermarkets per year in 2013–2017. With 27.2 million UK households in 2017, this amounts to around 51 unit purchases per household per year – or just less than one per week. Thus, these foods purchased from these supermarkets are only a relatively small component of household food consumption. We also found that purchases of common checkout foods have increased over time. This could be due to a growing snack food market, because people are shifting to smaller package sizes, or because supermarkets are gaining market share of common checkout foods from other retailers.

## Conclusions

Overall, older people purchased more common checkout foods than younger people, but there were no differences in purchases between those in most affluent versus other social grades. We found 14% fewer purchases of common checkout foods from supermarkets with clear and consistent checkout food policies compared to supermarkets with no policy, in the context of a crude background rate of purchasing of just less than one unit per household per week. There were no differences in purchases from supermarkets with vague or inconsistent policies compared to no policy. These differences in purchasing associated with clear and consistent checkout food policies were most pronounced in households with older main shoppers and those from the most and least affluent social grades. Given that more affluent and older people tend to have healthier diets, the social grade and age trends associated with clear and consistent policies are unlikely to ameliorate existing socio-economic age related inequalities in diet. Low-agency population interventions can only be expected to increase health equity when baseline inequalities disadvantage the least healthy.
